# Meta-synthesis about man as a father and caregiver for a hospitalized child

**DOI:** 10.1590/1518-8345.1850.2922

**Published:** 2017-09-18

**Authors:** Susana Maria Garcia dos Reis, Ana Carolina Andrade Biaggi Leite, Willyane de Andrade Alvarenga, Jeferson Santos Araújo, Márcia Maria Fontão Zago, Lucila Castanheira Nascimento

**Affiliations:** 1 Undergraduate student in Nursing, Escola de Enfermagem de Ribeirão Preto, Universidade de São Paulo, PAHO/WHO Collaborating Centre for Nursing Research Development, Ribeirão Preto, SP, Brazil.; 2 Doctoral student, Escola de Enfermagem de Ribeirão Preto, Universidade de São Paulo, PAHO/WHO Collaborating Centre for Nursing Research Development, Ribeirão Preto, SP, Brazil. Scholarship holder at Coordenação de Aperfeiçoamento de Pessoal de Nível Superior (CAPES), Brazil.; 3 PhD, Researcher, Universidade do Estado do Pará, Belém, PA, Brazil.; 4 PhD, Associate Professor, Escola de Enfermagem de Ribeirão Preto, Universidade de São Paulo, PAHO/WHO Collaborating Centre for Nursing Research Development, Ribeirão Preto, SP, Brazil.

**Keywords:** Fathers, Child Hospitalized, Masculinity, Qualitative Research, Pediatric Nursing

## Abstract

**Objective:**

to identify, analyze and synthesize the father’s experience in care for a hospitalized child from results of primary qualitative studies.

**Method:**

this is a qualitative meta-synthesis through which 12 articles were analyzed, selected in the Cumulative Index to Nursing and Allied Health Literature databases, Latin American and Caribbean Literature in Health Sciences, Public Medline, Scopus, PsycINFO and Web of Science, published between 1995 and 2015. The methodological steps proposed by Sandelowski and Barroso were used to systematize the review, as well as concepts from the anthropology of masculinities to analyze and discuss the synthesis.

**Results:**

the synthesis was presented by means of two themes: 1) paternal dilemmas - what man feels and faces during the hospitalization of the child, highlighting the emotional involvement and change in the family and work relationship, and 2) paternal identities - masculinities readjusted in view of the child’s illness, which reveals identity marks and repressed fatherhood in the hospital environment. Both themes illustrate the challenges and readjustment of parental identity.

**Final considerations:**

to get to know the experiences of the father during the hospitalization of the child and the way in which the challenges for the readjustment of roles related to masculinity could broaden the range of nursing and other health professionals, alerting to the importance of including the father as a protagonist or coadjuvant in the care for hospitalized children.

## Introduction

Although the differences between the concepts of fatherhood and motherhood are interpreted in various ways, in some cultures, the identity of the child’s caregiver, historically assumed by the woman, seems to be a universal consensus when analyzed from the hegemonic perspective of masculinity^1^ ^-^ ^2^. Masculinities multiply in the cultural universe of men though, and certainly coexist among the established social power structures; thus, complicity, subordination, marginalization, among others, are also power relations present in the paternal identities.

As for the care provided during the child’s illness, even when the primary caregiver is the woman, the family is reorganized the roles are redistributed among its members in view of the child’s illness and hospitalization^3^. At this stage, a range of feelings integrate the identity of the father, such as love, responsibility, concern, fear, anxiety, stress, guilt, sadness, impotence, helplessness and uncertainty regarding the child’s improvement^3^ ^-^ ^4^.

The distribution of identity roles in the traditional family, in which man is acknowledged as the provider and the woman as the caregiver, has been modified over the years^5^. This change is mainly due to social and economic changes, with emphasis on women spending more time outside their home, and culminating in the redefinition of the father’s participative roles in child care^5^.

At present, researchers have pointed out that, when the child is hospitalized, the father assumes the care for healthy children and domestic activities, while at the same time developing work activities to provide for the family, while the mother is responsible for accompanying the hospitalized child, giving up her daily activities^3^. Even when health professionals value the presence of the father as a caregiver in the hospital context^6^, the mother still has the role of the child’s primary caregiver^3^.

What the social aspect is concerned, the presence of the father as a caregiver in the hospital has been little observed, as has the incorporation of this identity by the fathers. Thus, in addition to the emotional factor, other impacts fall on the mother due to the child’s hospitalization, such as overload and concern with the domestic routine, the family at home, the sick child and the context of the hospitalization^3^.

When the father assumes the role of caregiver, a positive contribution is observed for the whole family, mainly concerning the physical, emotional, intellectual and social development of the child^7^. The man shows difficulty in assuming this role though, and considers himself to be an adjunct to the woman in this role, believing that she performs it better^4^.

The male hegemonic identities men have assumed in history as fathers of traditional Western families help to understand why they advocate the reproduction of behavioral stereotypes that do not fit the caregiver role. In diverse cultures, taking care of the children is interpreted as the incorporation of a hypo-male or feminine identity that is not compatible with the power and dominion exercised by the patriarchal man^8^. Thus, men distance themselves from care and approach the valuation practices of their protective, procreative, heterosexual and virile identity^2^.

Knowledge about parental practices in care for the hospitalized child is still scarce when compared to studies focused on the mother’s experience as a caregiver^9^. A search was conducted in the Cochrane Library to identify potential qualitative reviews about the father’s care for the hospitalized child, or the analysis of this care from the father’s own perspective. No review was identified, however, that emphasized the results of qualitative research focused on the father’s care for the hospitalized child, independently of the hospitalization sector, or the analysis of this care from the paternal perspective.

Different literature reviews have evaluated the experiences of the father of children with cancer^10^ and type 1 diabetes^11^, of the father with newborns admitted to Neonatal Intensive Care Units (NICU)^12^, of the father during the first year of the child’s life^13^, his contribution to managing the child’s chronic condition^14^, his participation in the child’s hospitalization^15^, and the different role perceptions between Eastern and Western parents in view of crisis situations related to the child’s illness^16^. The lack of reviews on the experience of the caregiver father of a hospitalized child expresses the importance of synthesizing the current knowledge from qualitative studies based on the perspective of the father who experiences the hospitalization of the child with different clinical conditions. The interpretation and synthesis of these qualitative data are crucial aspects to identify the direction of future studies, to maximize the father’s experience, besides appointing the father’s needs in care for the hospitalized child.

The review question was “What has been the paternal experience in care for hospitalized children?”. The objective of this review was to identify, analyze and synthesize the father’s experience in care for the hospitalized child from results of primary qualitative studies.

## Methods

This is a qualitative meta-synthesis. The approach used to develop it involved the following steps, proposed by Sandelowski and Barroso^17^: a) elaboration of the research question and problem, b) systematic identification and selection of articles for analysis, c) quality appraisal of articles, d) extraction of the data and e) elaboration of the synthesis. Recommendations described in ENTREQ (Enhancing transparency in reporting the synthesis of qualitative research) were used to report the qualitative synthesis^18^.

We started with a comprehensive literature search to identify all articles that used a qualitative method to describe the experience of the father in care for the hospitalized child. The search was performed by two reviewers, independently, in the databases CINAHL, LILACS, PubMed, Scopus, PsycINFO and Web of Science. The following combination of descriptors and keywords was used to conduct the search in the PubMed database, as well as in the other databases, with slight adaptations (except in LILACS), according to their particularities: “father” [descriptor] OR “fathers” [keyword] OR father [keyword] AND “child, hospitalized” [descriptor] OR (“child” AND “hospitalized”) [keyword] OR “child hospitalized” [keyword] AND “father-child relations” [descriptor] OR (“father-child” AND “relations”) [keyword] OR “father-child relations” [keyword] OR (“father” AND “child” AND “relations”) [keyword]; with the following data limits [01/01/1995 till 12/31/2015]; species [humans] and language [Portuguese, English or Spanish]. In LILACS, the following search strategy was used: “pai” [descriptor] AND “relações pai-filho” [descriptor] OR (“relações” AND “pai-filho”) [keyword] AND “criança hospitalizada” [descriptor] OR (“criança” AND “hospitalizada”) [keyword].

Articles published between 1995 and 2015 were included, in English, Portuguese and Spanish, using a qualitative method, focused on the father’s experience, reporting on his perspective on the care for hospitalized children between 0 and 18 years of age. Articles that reported on the perspective of the father and the mother were also included if the results related to the father were presented separately from those of the mother. It was decided to exclude theses and dissertations, abstracts published in congress annals, editorials of journals, literature reviews and articles on the experience of the father from the perspective of other relatives of the child or health professionals. The database search occurred in May 2015 and was updated in January 2016.

**Figure 1:**
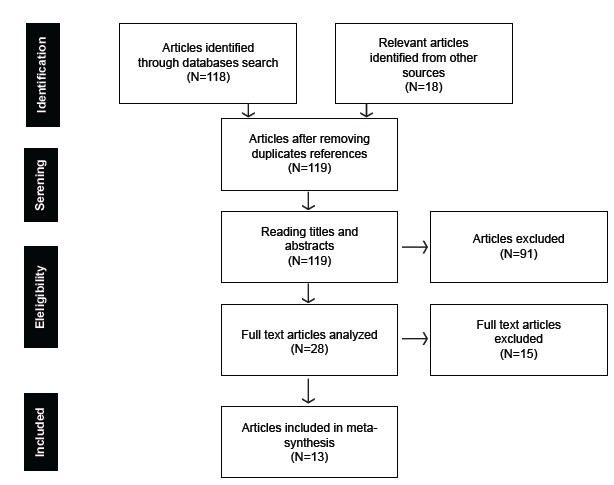
PRISMA flowchart of literature search process

Figure 1 illustrates the search process in the literature and its description followed the PRISMA recommendations^19^ to report on the inclusion process of the studies. In total, 136 references were obtained, of which 118 were identified in the databases and 18 in other sources, derived from the personal collection of the authors of this meta-synthesis (N = 2), from the references of the included articles (N = 1) and from relevant literature reviews on parental care (N = 15)^10^ ^-^ ^16^. A total of 119 articles were reached after the removal of repeated references. Two reviewers read the titles and abstracts of these articles and judged, independently and later together, if the articles met the inclusion criteria. As a result of this process, 28 articles met the eligibility criteria. To evaluate the interobserver agreement, the Kappa coefficient^20^ was calculated, and the result obtained indicated 0.943, which represents almost perfect agreement. Then, both reviewers independently read the full texts of the 28 articles and, with the help of a third reviewer, experienced in qualitative studies and in the study subject, determined the final eligibility of the articles. After this step, 15 articles were excluded due to the research method (N = 5), because these articles did not focus on the experience of the parent as caregiver (N = 7), because the article was a summary (N = 1) and because the child was not hospitalized (N = 2), which resulted in a final sample of 13 articles. 

The quality of the studies was evaluated through the CASP (Critical Appraisal Skills Program)^21^, an instrument that consists of a checklist for the evaluation of qualitative research. The instrument is composed of 10 questions, two of which screen the applicability of the instrument to the article and eight deal with study design, recruitment and data collection strategy, reflexivity, ethics, data analysis, results and implications of qualitative studies. Two reviewers independently assessed the studies, based on the criteria listed above, and discussed the differences between their assessments to achieve agreement. It was not considered relevant to exclude any of the 13 studies based on quality, since this meta-synthesis is focused on a problem in a knowledge are under development and, therefore, all were considered important, because they contribute to the understanding of the father’s experience in care for the hospitalized child. Another reason was the lack of agreement on whether or not to include studies on the basis of structured approaches to the assessment of research quality^22^.

In the process of extracting and synthesizing the data, first, two reviewers carried out successive readings of the 13 complete articles, from which they extracted the data independently. According to the review question, the data were organized in a standardized form, developed by the reviewers for that purpose. Information about the method, study participants, and results was extracted by thoroughly evaluating articles, line by line. Secondly, together, the reviewers re-evaluated all the data extracted, reaching a consensus on divergences in the initial evaluation.

Simultaneously with the data extraction, coding was performed to elaborate the categories related to the experience of the father in care for the hospitalized child, in order to facilitate the synthesis of the data. The process of coding the results of the articles was guided by the thematic analysis, composed of 6 phases: i) get familiar with the data, ii) generate the initial codes, iii) search the themes, iv) review the themes obtained, v) define and name the themes and vi) produce the final report^23^. Coding was performed inductively, in that codes were continuously compared and related. Subsequently, the reviewers independently organized the codes in descriptive themes and then together, aiming to solve some conflict, interpret them critically and develop the analytical themes. To complete the synthesis and maximize validity, a third reviewer integrated the team and carefully checked the fit of the codes in each category, as well as the concepts related to the categories listed. The themes were carefully integrated and expanded to determine a general conceptualization of the data and, to this end, concepts from the anthropology of masculinities^(1-2, 24)^ were used to explain the constructed themes. Thus, a new interpretation was possible for the results of the primary studies.

## Results

**Figure 2:**
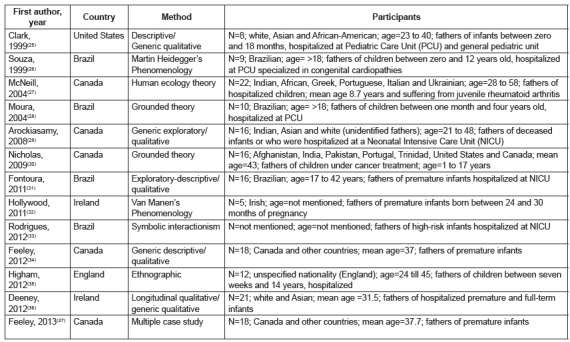
Main characteristics of included studies. Ribeirão Preto, SP, Brazil, 1995-2015

The included studies (n = 13) were developed in Canada (n = 5), Brazil (n = 4), United States (n = 1), England (n = 1) and Ireland (n=2), as shown in Figure 2. The 13^25^ ^-^ ^37^ ^)^ articles included a total of 171 fathers, over 18 years old and of different ethnic origins, who experienced the hospitalization of their son/daughter in intensive care settings (n = 7) and pediatric wards (n = 6). Several qualitative designs were used, including qualitative and interpretative description (n = 3), exploratory (n = 1), exploratory and descriptive (n = 1), grounded theory (N = 1), ethnography (n = 1), case study (n = 1) and symbolic interactionism (n = 1). In five^25^ ^,^ ^27^ ^,^ ^29^ ^,^ ^31^ ^,^ ^34^ studies, the theoretical framework that supported the development of the study was not presented. These articles were named in the present study as generic qualitative^38^, since they were not guided by an explicit or established set of philosophical presuppositions, in the form of a known qualitative method, such as the grounded theory for example^26^ ^,^ ^28^.

**Figure 3:**
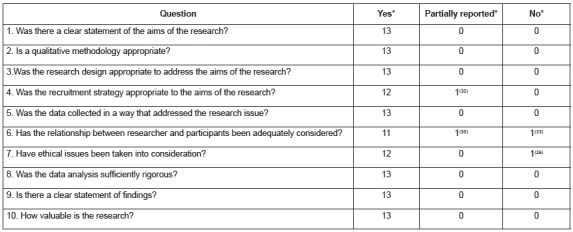
Qualitative assessment of included studies according to Critical Appraisal Skills Programme (CASP). Ribeirão Preto, SP, Brazil, 2016

The quality of the qualitative studies was generally considered to be good, according to Figure 3. Most of the studies^25^ ^-^ ^29^ ^,^ ^31^ ^-^ ^32^ ^,^ ^34^ ^-^ ^37^ (n = 10) were judged to appropriately report all questions on the CASP checklist^21^. In one of the studies^26^, there was no mention of ethical aspects. In another^30^, the information about the recruitment strategy used and the relationship between the researcher and the participants were not adequately reported. And, in a third study^33^, the information about the relationship between the researcher and the participants was not mentioned.

**Figure 4:**
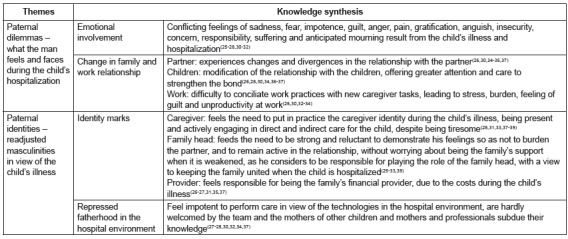
Themes elaborated by means of the analysis, integration and interpretation of the findings of the included articles

The study findings were explored and two main themes could be elaborated through the meta-synthesis: a) paternal dilemmas - what the man feels and faces during the child’s hospitalization and b) paternal identities - readjusted masculinities in view of the child’s illness. These themes are presented in Figure 4 and illustrate the father’s perspective in care for the hospitalized child.

### Paternal dilemmas - what the man feels and faces during the child’s hospitalization

This theme represented the dilemmas related to the repercussions of the child’s hospitalization on the paternal emotional state and on their family and work relations. For parents, hospitalization is considered a moment of devastating crisis for the whole family^27^ ^-^ ^28^ ^,^ ^30^. This phase is experienced with a variety of feelings, in which the bond and joy of becoming a father are threatened by the challenge of dealing with the child’s vulnerability and possibility of death^26^ ^,^ ^33^. Among the conflicting feelings are guilt, anger, pain, sadness, insecurity, worry, responsibility and fear of the future^26^ ^-^ ^28^ ^,^ ^31^ ^-^ ^34^. All of them derive from the context of the child’s illness and are capable of affecting the father’s organism, who starts to present insomnia, anxiety, feeling of having a dry throat and lack of appetite^31^.

The hospitalization of the child also affects the relationships with the partner. Studies have shown the need to negotiate functions between the couple to meet the demand for care of the sick child and the family^27^ ^,^ ^36^. In this process, divergences usually emerge when the mother directs her attention only to the hospitalized child, which affects the couple’s relationship^31^. In addition, at the moment when she acts as an inspector, she directly and indirectly influences the father’s involvement in the care for the hospitalized child, to the point of hindering his insertion in this function^35^ ^,^ ^37^. This maternal influence on parental care is also associated with the father’s insecurity in dealing with the situation, which leads him to behave as a coadjuvant of the partner^27^ ^,^ ^32^ ^,^ ^36^, requesting her approval for any decision on the care for the sick child^27^ ^,^ ^36^.

Regarding the relationship with the children, the hospitalization made the fathers afraid of engaging in care out of fear of harming them^26^ ^,^ ^32^ ^,^ ^35^ ^,^ ^37^, and also because of the technologies and barriers of the hospital space, which hindered their involvement, especially in situations where twins were hospitalized in distant beds^35^ ^,^ ^37^. Parents identified positive changes in their relationships with their children after the illness^27^ ^-^ ^28^ ^,^ ^31^ though, acknowledging the importance of being present in the recovery process^29^ ^,^ ^35^ ^,^ ^37^. The situation of crisis made them more involved in parenting^37^, and when involvement in care culminated in the improvement of the children’s clinical condition, feelings of happiness and love emerged^29^ ^,^ ^37^.

With the son’s illness, new demands arise for the family; the father needs to support his wife emotionally, perform household chores, and care for other children, while at the same time financially providing the family with work, which creates high levels of stress^(31, 34-35)^. The father, because he is considered the provider, needs to work and show productivity at work precisely at the moment when he would also like to be present in the hospitalization of the child^28^ ^-^ ^30^, or alongside other family members^27^ ^-^ ^28^ ^,^ ^31^. This impossibility of becoming more involved in the care for the child and the family, as well as the need to attend to the work activities, generates feelings of guilt that affect their productivity^28^ ^-^ ^30^ ^,^ ^32^. Studies have pointed out that men want flexible work hours to meet these demands; parents from higher economic classes are more successful in this negotiation though^31^ ^,^ ^37^.

### Paternal identities - readjusted masculinities in view of the child’s illness

Identity marks of masculinities were evidenced with the son’s illness, and the fathers in the studies analyzed reported the feeling of repressed fatherhood in the hospital environment. Some showed that the presence of the man in the hospital generates discomfort for him, for other companions and for the team^28^ ^-^ ^29^ ^,^ ^32^ ^,^ ^37^. He does not feel welcomed by health professionals and other companions of children in the pediatric unit, since it is a space predominantly attended by mothers and, therefore, it is their social function to accompany the child’s hospitalization process. The fathers indicated that this environment is not prepared to welcome them^(28-29, 37)^.

In addition to not being accepted in the hospital context, the man feels judged by the other mothers because he does not have the same knowledge and the same abilities expected from the mother figure, which makes him feel sad and helpless^27^ ^,^ ^31^. Linked to this, he considers that health professionals can hamper the care he provides, limiting his access to information by using technical language^27^ ^,^ ^29^ ^-^ ^30^ ^,^ ^33^ and making it difficult for him to take control of the situation^30^ ^,^ ^35^ ^,^ ^37^. He also feels a lack of support in the hospital environment and believes that the mother receives such support^30^ ^,^ ^32^ ^,^ ^37^.

With the son’s illness, studies have pointed out that the father tries to assume his role of caregiver by being present and involved in caring for the child’s needs, even if, in order to do so, he has to neglect his own needs^26^ ^,^ ^29^ ^,^ ^31^ ^,^ ^36^ ^-^ ^37^. In order to assert his masculinity, being head of the family, he is healthy and strong in front of the family^26^ ^-^ ^28^ ^,^ ^30^ ^-^ ^31^ ^,^ ^36^ ^-^ ^37^ and is reluctant to show his feelings, so as not to burden the partner, since he considers her fragile^26^ ^-^ ^28^ ^,^ ^30^ ^-^ ^31^ ^,^ ^37^. There is a feeling of helplessness and sadness though because of the inability to protect the hospitalized child and his family^30^ ^-^ ^31^.

The father also has a sense of lack of control as he is unable to protect the child from pain and suffering, as well as to maintain the stability of the family, because he cannot meet the entire demand deriving from the illness^26^ ^,^ ^30^ ^-^ ^31^. It is through work that man tries to recover his hegemonic masculinity, maintaining himself as provider of the family to mitigate the financial impact caused by the disease^26^ ^,^ ^30^ ^-^ ^31^. The father seeks, at all times, to make decisions that keep his family together, trying to reassume his role as protector^27^ ^-^ ^28^ ^,^ ^31^ ^,^ ^36^ ^-^ ^37^. In this attempt, he also assumes the responsibility for guaranteeing the quality of care, defending the health needs of his son^27^ ^-^ ^28^ ^,^ ^31^ ^,^ ^36^ ^-^ ^37^.

The fathers demonstrated overconfidence in their self-control to mediate the situation, but despite their reluctance to accept support, they made it clear that they needed help^28^ ^,^ ^31^. Other ways of seeking control were: escape from the situation with physical activity, search for positive meanings for the child’s illness and support beliefs, maintenance of hope and resumption or adoption of spiritual practices^27^ ^-^ ^28^ ^,^ ^30^ ^-^ ^31^. Studies show that, in the paternal perspective, the child’s illness brings personal growth to the man^27^ ^-^ ^28^ ^,^ ^34^ and to those around him^30^.

## Discussion

This meta-synthesis permitted a rigorous review of the qualitative literature and also analyzed the father’s experience in care for the hospitalized child, from the paternal perspective, enabling the identification of factors that characterize the care of the father to the hospitalized child and the influence of this context in the scope of their family relations and work, as well as in the readjustment of their paternal identity. Because studies refer to the identity nature of masculinities in their results, concepts from the anthropology of masculinities^(1-2, 24)^ have provided an appropriate approach to assign meanings to the paternal experience and the elaboration of the qualitative synthesis. When a cultural approach is given to male behaviors^1^, man is perceived as the product of his social environment, and the effects of his behaviors are justified by gender patterns and masculinities that fit each culture^24^. Thus, paternal behavior, during the illness of the child, is understood as influenced by the way in which the fathers experience the culture, masculinities and gender.

Studies have shown that seeing his child ill triggers a series of emotions in the father^26^ ^-^ ^28^ ^,^ ^31^ ^-^ ^34^ because, as highlighted, when the child becomes ill, the whole family becomes ill together^27^ ^-^ ^28^ ^,^ ^31^. The father’s emotional involvement is characterized as a posture that leads him to try to escape from the male stereotypes that imply that he is strong, contains his emotions and does not care for others^12^. These masculine identity norms, acquired from the cultural midst, collide with the new needs of the family, since the father adopts new identities, like that of caregiver^31^. He also assumes behaviors historically delegated to the feminine, like that of caregiver, and understands that they can be equally shared by man. There is, therefore, a redefinition of the hegemonic masculinity identity towards another, which is anchored in the concept of multiple masculinities, since men, as well as the culture that governs them, assume identity roles that vary according to their historical time, social class and experience they acquire throughout their lives^1^ ^,^ ^8^.

Regarding the paternal masculinities, evidenced in the literature analyzed^27^ ^,^ ^36^, specifically the identity of care approached the participants in the studies to a subordinate masculinity, so that they reacted with concern towards the illness and responsibility for the care of the hospitalized child, culturally delegated to the female gender^1^ ^-^ ^2^, which therefore fits them into a subordinate masculinity. The subordinate masculinity refers to the identity in which the man submits to a situation of being dominated by a hegemonic pattern^1^. In this study, the fathers were subordinated to the women’s domain, due to their insecurities to make decisions regarding child care without the prior approval of the mothers^27^ ^,^ ^36^.

The meta-synthesis showed that the child’s illness interfered in the father’s marital relationship, since there is the initial impact of the child’s illness, uncertainties about the future, and the need to make new decisions about the child’s treatment^27^ ^-^ ^28^ ^,^ ^31^. Father and mother are frightened by the possibility of loss, generating an imbalance in the couple’s relationship and the need for greater support^30^ ^-^ ^31^. The negotiation of the roles occurs to attend to several other family activities, while at the same time it is necessary to provide the family with financial support through work^27^ ^,^ ^32^ ^,^ ^36^. Such behaviors put the fathers in a relationship of complicity, in which the power exercised by their masculine identities on the woman does not keep them in a relationship of subordination, but of complicity, by sharing functions culturally attributed to the female gender.

The masculinity of complicity presents itself as one in which some precepts of patriarchy are shared, but there is no full adoption of hegemonic patterns, which places men in an identity of accomplice of various behaviors culturally associated with the feminine gender^1^ ^-^ ^2^.

Throughout the child’s illness, this meta-synthesis has shown that the woman reaffirms her role as a caregiver and may resist allowing the father to share this role^35^ ^,^ ^37^. When the father shows the ability to do so, the mother is receptive to his care, but when he shows insecurity, she resists allowing his involvement. This indicates how the father needs the support of the mother to participate more effectively in care for sick children, since this support can motivate him and make him feel safe to better carry out the activities related to fatherhood, adapting to the role of caregiver.

Even in the hospital environment, the father faces a range of conflicting situations by establishing himself as the child’s caregiver. He does not feel welcomed in the hospital by staff members and other caregivers of children, mostly mothers^27^ ^-^ ^32^ ^,^ ^37^. These results agree with what has been pointed out in the literature^6^. It is perceived that the different masculinities are in constant disputes with each other, as well as the cultural identities that produce them^1^. Promoting the adoption of male behaviors, deviant from hegemonic patterns, contributes to the development of new identities, one of which is revealed by paternal care in the hospital space^39^ ^-^ ^40^. Although this act calls for adaptations that restrain hegemonic patterns of masculinity, such as non-sensitivity and relations of dominance over women, parents feel insecure and have shown fear of harming the child in situations of fragility due to illness^26^ ^,^ ^32^ ^,^ ^35^ ^,^ ^37^. The unfriendly hospital environment, with innumerable technological devices, and the impossibility of touching or holding the child^32^ ^,^ ^35^ ^,^ ^37^ were other triggers of the fathers’ insecurity.

Even with these barriers to care, the father were able to identify positive changes in the relationship with their children after the illness^27^ ^-^ ^28^ ^,^ ^31^, as they became more involved in caring and became closer to the child. The father acknowledges his importance in the recovery of his child^29^ ^,^ ^32^ ^,^ ^35^ ^,^ ^37^ and wants to be a better father^37^. Their presence has essential effects for the development of the child, who needs the support, security and values transmitted in this relation^14^.

Literature also points to the emergence of new masculinities for the father in the face of social changes as, besides continuing to serve as the family provider, he is expected to take care of the child, along with the partner, in a more flexible, affectionate and egalitarian way^41^. Therefore, being a man, father and caregiver of a hospitalized child corresponds to the adoption of an identity that is in constant process of redefinition of masculine roles. The field of pediatric health perceives the father as a potential caregiver and partner in child care, even if this role does not integrate the repertoire of concerns surrounding culturally established hegemonic masculinity, including in health services.

## Limits and strengths

The results of this meta-synthesis should be considered in the context of its limitations, as the sample of 13 articles may have restricted the range of the phenomenon studied. For example, the included studies pictured nuclear families made up of father, mother and children, which does not reflect the complexity of the man as the father and caregiver of a hospitalized child, like in the case of a homosexual relationship. Although the review has included articles from different countries, such as Canada, Brazil, the United States, England and Ireland, it still does not represent a global perspective on the father’s care for the hospitalized child in different cultures. The lack of demographic data and the limited information on the characteristics of participants in the studies included did not permit a more detailed analysis. The process of interpreting the results based on the concepts of the masculinity anthropology, however, broadened and strengthened the comprehensive explanation for the elaborated themes, which represent the experience of the man, father and caregiver of the hospitalized child. The meta-synthesis also assessed the quality of the studies through the CASP checklist and pointed out the qualitative shortcomings in the presentation of the studies, alerting researchers to the need to improve the essential elements in the presentation of the qualitative studies, in order to value the rigor in their conduction, analysis and in the application of the results.

## Final considerations

In this meta-synthesis, the father’s perspective in child care during the hospitalization process was presented. The man engages emotionally and faces changes in family and work relationships. He frequently readjusts his masculinity to meet the demands and expectations, adapting to his new functions and reality, not always supported by the wife and the health team in this process.

The presentation of this meta-synthesis is particularly important in nursing. It supports the design of new studies, particularly the need to present the perspective of the father separately, when other figures of parenting are included, with a view to permitting the analysis or comparison of their perspectives. The importance of presenting the socioeconomic situation, the condition of life, the culture and the family composition of the father is reiterated, so that they are related to the particularities of the paternal experiences. It is also suggested that new studies incorporate diverse family structures, seeking the experience of other figures who assume the role of father (stepfather, uncle, grandfather and others), in the context of the child’s hospitalization. Finally, it is suggested to use the anthropological perspectives on culture and masculinities for the analysis of future research data.

The results also contribute to the planning of nursing care, articulated to the health team and family, with a view to the adaptation and better performance of the paternal functions in the hospital context. Knowing the experiences of the father during the hospitalization of the child, as well as the difficulties for the readjustment of masculinity roles, could increase the importance of including him in the context of hospitalized child care, as a protagonist or coadjuvant, with a view to qualifying nursing care.
